# Dual-Task vs. Single-Task Gait Training to Improve Spatiotemporal Gait Parameters in People with Parkinson’s Disease: A Systematic Review and Meta-Analysis

**DOI:** 10.3390/brainsci14050517

**Published:** 2024-05-20

**Authors:** Elisabetta Sarasso, Marco Pietro Parente, Federica Agosta, Massimo Filippi, Davide Corbetta

**Affiliations:** 1 Vita-Salute San Raffaele University, 20132 Milan, Italy; sarasso.elisabetta@hsr.it (E.S.); marcopietro.parente@gmail.com (M.P.P.); agosta.federica@hsr.it (F.A.); filippi.massimo@hsr.it (M.F.); 2 Neuroimaging Research Unit, Division of Neuroscience, IRCCS San Raffaele Scientific Institute, 20132 Milan, Italy; 3 Department of Neuroscience, Rehabilitation, Ophthalmology, Genetics and Maternal Child Health, University of Genoa, 16132 Genoa, Italy; 4 Neurology Unit, IRCCS San Raffaele Scientific Institute, 20132 Milan, Italy; 5 Neurorehabilitation Unit, IRCCS San Raffaele Scientific Institute, 20132 Milan, Italy; 6 Neurophysiology Service, IRCCS San Raffaele Scientific Institute, 20132 Milan, Italy; 7 Department of Rehabilitation and Functional Recovery, IRCCS San Raffaele Scientific Institute, 20132 Milan, Italy

**Keywords:** Parkinson’s disease, physical therapy modalities, gait analysis, meta-analysis

## Abstract

Background: People with Parkinson’s disease (pwPD) present alterations of spatiotemporal gait parameters that impact walking ability. While preliminary studies suggested that dual-task gait training improves spatiotemporal gait parameters, it remains unclear whether dual-task gait training specifically improves dual-task gait performance compared to single-task gait training. The aim of this review is to assess the effect of dual-task training relative to single-task gait training on specific gait parameters during dual-task tests in pwPD. Methods: We conducted a systematic review and meta-analysis of randomized controlled trials (RCTs), searching three electronic databases. Two reviewers independently selected RCTs, extracted data, and applied the Cochrane risk-of-bias tool for randomized trials (Version 2) and the GRADE framework for assessing the certainty of evidence. The primary outcomes were dual-task gait speed, stride length, and cadence. Secondary outcomes included dual-task costs on gait speed, balance confidence, and quality of life. Results: We included 14 RCTs (548 patients). Meta-analyses showed effects favoring dual-task training over single-task training in improving dual-task gait speed (standardized mean difference [SMD] = 0.48, 95% confidence interval [CI] = 0.20–0.77; 11 studies; low certainty evidence), stride length (mean difference [MD] = 0.09 m, 95% CI = 0.04–0.14; 4 studies; very low certainty evidence), and cadence (MD = 5.45 steps/min, 95% CI = 3.59–7.31; 5 studies; very low certainty evidence). We also found a significant effect of dual-task training over single-task training on dual-task cost and quality of life, but not on balance confidence. Conclusions: Our findings support the use of dual-task training relative to single-task training to improve dual-task spatiotemporal gait parameters in pwPD. Further studies are encouraged to better define the features of dual-task training and the clinical characteristics of pwPD to identify better responders.

## 1. Introduction

Parkinson’s disease (PD) is the second-most common neurodegenerative disease after Alzheimer’s disease [1]. Beyond bradykinesia/akinesia, tremor, and rigidity, people with PD (pwPD) are often characterized by significant alterations of spatiotemporal gait parameters that often affect walking ability [2]. Such an impairment in walking is exacerbated when pwPD are concurrently engaged in dual-task activities involving the simultaneous execution of motor and cognitive functions. Accordingly, a substantial body of evidence suggests that the spatiotemporal gait parameters deteriorate concomitantly with attention, memory, information processing, visuospatial, or verbal fluency tasks [3,4]. Several studies have demonstrated that engaging in dual-task activities also increases the risk of falls in pwPD [5]. Moreover, pwPD exhibit diminished dual-task performance when compared to age-, sex-, and education-matched healthy controls [6].

Dual-task training, despite past controversies about potential risks [7], represents a promising approach in the treatment of pwPD. Dual-task gait training consists of combining a walking activity with another cognitive or motor activity at the same time. The goal of this approach is to improve the patient’s ability to coordinate motor and cognitive functions, often compromised in pwPD. Dual-task gait training can promote brain plasticity and favor the adaptation and reorganization of the neural networks involved in movement control [8,9]. This training operates by enabling patients to enhance their motor automaticity, defined as the ability to execute a learned movement without requiring excessive attentional control [10].

Recently, several studies suggested that dual-task gait training could lead to improvements in gait speed and stability, balance, and motor control in pwPD over time [11,12,13,14,15]. Focusing on task-specific interventions can indeed be crucial for targeting particular aspects of gait performance. When it comes to the dual-task impact on gait quality, where individuals are required to simultaneously perform cognitive tasks while walking, tailored training regimens can potentially yield more targeted improvements compared to gait training alone. However, none of the recent systematic reviews has analyzed the effect of dual-task compared to single-task gait training on the improvement of spatiotemporal gait parameters during dual-task performance.

In order to fill this gap, the primary aim of this review was to compare the effects of dual-task gait training in comparison to single-task gait training to improve spatiotemporal parameters of speed, stride length, and cadence during dual-task gait/mobility in pwPD.

## 2. Materials and Methods

The protocols were registered with Protocols.io (dx.doi.org/10.17504/protocols.io.36wgq314olk5/v1, accessed on 15 May 2024) according to the Preferred Reporting Items for Systematic Reviews and Meta-Analyses (PRISMA) [16].

### 2.1. Search Strategy

We conducted this review and meta-analysis following the PRISMA [16]. We searched for trials in English in the following electronic bibliographic databases on 30 June 2023: PubMed, EMBASE, and the Cochrane Central Register of Controlled Trials (CENTRAL). A search strategy was developed including terms related to the population, ‘Parkinson’s disease’, and the intervention ‘dual-task gait training’ in combination with the sensitivity-maximizing version of the Cochrane highly sensitive search strategy to identify randomized controlled trials (RCTs) [17]. Handsearching was also carried out by consulting the references to the included studies to look for any further trials that could be included. The search strategy was adapted to each bibliographic database (See Supplementary File S1, Search strategies).

### 2.2. Inclusion Criteria

We included studies enrolling adults with idiopathic PD receiving dual-task (cognitive) gait training alone or added to single-task gait training to improve gait speed, stride length, and gait cadence. Dual-task (cognitive) gait training is the repetitive practice of any intervention involving gait training (either on a walking ground or treadmill) with a concurrent cognitive task that allows to stimulate both motor and cognitive processes. The trials included comparing dual-task gait training to active intervention (separate practice of motor and cognitive tasks, upper limb training, and balance exercises) or inactive intervention (education, no treatment), but only trials in which the control group underwent single-task gait training were used as a comparator in the meta-analyses. Other exclusion criteria were the following: (1) multi-modal interventions, (2) absence of dual-task gait parameter measurements in the outcome assessment, (3) training combined with invasive or non-invasive brain stimulation techniques, (4) single session studies.

Primary outcomes were dual-task spatiotemporal gait parameters: gait speed expressed as meters per second, stride length expressed in meters, and cadence expressed in steps per minute. Secondary outcomes were dual-task cost on gait speed, expressed as the ratio of the difference in speed from dual-task to single-task over the dual-task speed per 100 or the single-task speed per −100, balance confidence, and quality of life.

### 2.3. Study Selection

Retrieved citations were imported into Rayyan (an AI powered tool for systematic literature reviews) [18]. We used the ‘find duplicates’ facility of Rayyan to detect and remove duplicates. Citations were also manually cross-checked by one author to detect duplicates. Two reviewers independently (MPP and ES) screened the title and abstract to identify citations eligible for inclusion and assessed the full text of potentially eligible studies according to the inclusion and exclusion criteria. In the case of disagreement, a third author (DC) was consulted to reach consensus. Reasons for exclusion were reported in the PRISMA flowchart (Supplementary File S1, Supplementary Figure S1).

### 2.4. Data Extraction and Management

Two authors (ES, MPP) independently extracted data using a standardized form, including the following information: (1) bibliographic information (title, authors, year of publication, sample size), (2) characteristics of participants (age, disease duration, severity of motor symptoms measured through the Unified Parkinson’s Disease Rating Scale [UPDRS] score part III, Hoehn and Yahr stage [H&Y]), (3) type of intervention, (4) type of comparison, and (5) outcomes reported. One author (MPP) extracted, for each of the included studies: sample size, outcome measures, and their relative values expressed as means and standard deviations (SDs) before and after the intervention period. We estimated unreported measures of variability according to the variances of similar studies [17] and followed the methods proposed by Wan et al. [19] to approximate means and SDs in the case of data with a non-parametric distribution.

### 2.5. Risk of Bias Assessment

Two reviewers (ES and MPP) independently assessed the risk of bias of the included studies using Version 2 of the Cochrane risk-of-bias tool for randomized trials (RoB 2) [20]. RoB 2 is an outcome-based evaluation that is structured into a fixed set of domains of bias, focusing on different aspects of trial design, conduct, and reporting. Within each domain, a series of ‘signaling questions’ aim to elicit information about features of the trial that are relevant to the risk of bias. A proposed judgement about the risk of bias arising from each domain is generated by an algorithm, based on answers to the signaling questions. The RoB 2 provides an overall judgement, based on domain-level judgement, that can be ‘Low’ or ‘High’ risk of bias, or can be expressed as ‘Some concerns’. In the case of a discrepancy in the assessment performed by the two reviewers, a third reviewer (DC) was consulted to reach consensus.

### 2.6. Data Synthesis and Analysis

To compare dual-task versus single-task gait training, we conducted meta-analyses using data from studies in which the comparison group performed single-task gait training. For trials comparing dual-task training to passive treatment or no treatment, we employed as a comparator the data from a single-task gait training group identified among the included studies with the lowest risk of bias and the most homogeneous in terms of clinical characteristics of participants, including age, Hoehn and Yahr score, and disease duration. To mitigate the unit-of-analysis error, the total number of participants in the selected single-task gait training group was divided by the number of comparisons [17]. The overall estimation of the effect was expressed as the mean difference (MD) with a 95% confidence interval (CI) when studies used a similar scale or measured the same outcomes. Standardized mean difference (SMD) with a 95% confidence interval (CI) was instead used for comparisons of different scales of outcome measurements. The effect size, in this case, was interpreted using Cohen’s criteria for pooled estimates. Cohen’s criteria measure the effect of a treatment with ≤0.2 as a small effect, 0.2–0.5 as a moderate effect, 0.5–0.7 as a large effect, and >0.7 as a very large effect. To assess the robustness of our estimations, we performed a sensitivity analysis by replacing the data of the comparison groups used in the meta-analyses with those of the other studies. We used STATA 16 software (StataCorp LCC, College Station, TX, USA, Release 16) [21]. The studies not included in the meta-analysis were reviewed and presented using a narrative approach.

### 2.7. Assessment of Heterogeneity and Subgroup Analysis

Heterogeneity was evaluated using a chi-squared test and using the *I*^2^ statistics following the interpretation of the Cochrane handbook: 0–40%: might not be important; 30–60%: may represent moderate heterogeneity; 50–90%: may represent substantial heterogeneity; 75–100%: considerable heterogeneity [22]. The use of a random effects model was chosen, regardless of the *I*^2^ level of heterogeneity, considering clinical heterogeneity. Subgroup analyses were performed according to disease severity. Since not all trials reported the H&Y values at the baseline, we used the UPDRS III baseline assessments to evaluate disease severity as suggested by Martìnez-Martìn et al. [23]. The authors proposed cut-off points to classify pwPD as mild, moderate, or severe based on their UPDRS score. We stratified trials into three categories of severity: mild (UPDRS III ≤ 32), moderate (32 < UPDRS III ≥ 58), and severe (UPDRS III > 58). Trials that did not report a UPDRS-III score at baseline were excluded from the subgroup analysis.

### 2.8. Quality of the Evidence

The Grading of Recommendation Assessment, Development, and Evaluation (GRADE) framework was used to assess the certainty of evidence for each outcome. The GRADE approach considered, for each outcome, four domains: (1) risk of bias; (2) inconsistency of the results; (3) indirectness; and (4) imprecision of results. The evaluation of the certainty of the evidence started from ‘high’ since only RCTs were included. Then, the level of evidence was downgraded for one or two levels according to: (i) the risk of bias (level downgraded if the risk of bias among studies was judged to be high); (ii) *I*^2^ score (level downgraded if the score was 50% or more); (iii) outcome measures (level downgraded if different outcome measures were used across studies to assess one outcome); (iv) confidence interval (level downgraded if the estimation included the null effect). Thus, the levels of evidence were progressively ‘high’, ‘moderate’, ‘low’, and ‘very low’.

## 3. Results

### 3.1. Flow of the Screening Process

The search identified 500 records (Supplementary File S1, Supplementary Figure S1). After duplicate removal and title/abstract screening, 47 full-text papers were assessed for eligibility. Therefore, only 14 studies met the inclusion criteria and were included in the review [9,24,25,26,27,28,29,30,31,32,33,34,35,36] (Supplementary File S1, Flow of the screening process—exclusion criteria).

### 3.2. Characteristics of Included Studies

Included studies were published from 2012 to 2022; 4 studies were multicenter across different countries (Israel, the United Kingdom, Italy, the Netherlands, and Belgium) [26,28,29,32], 4 studies were conducted in the USA [25,27,30,31], 2 in Sweden [24,34], 1 in Italy [9], 1 in Spain [33], 1 in China [35], and 1 in Taiwan [36].

### 3.3. Methodological Quality Assessment

The risk of bias of the included studies, including all domain judgements, analyzed through RoB 2, is graphically represented in Supplementary File S1, Supplementary Figure S2. Most trials were judged to be at high risk of bias or presented some concerns. The most common risks of bias among studies were: incomplete information on the randomization process and allocation concealment; risks of bias arising from the selection of the reported results; and the lack of published protocols. In fact, for some studies, the work protocol was unavailable. Furthermore, one study has raised the risk of bias for missing outcome data, and another study used a possible inappropriate measure for the ‘dual-task cost on gait speed’ outcome; consequently, the risk of bias has grown. In almost all studies, the participants and physiotherapists were not blinded to the treatment administered. However, this bias was partially compensated by the fact that there were no deviations from the intended intervention and that the studies used appropriate statistical analyses. However, in all 14 studies, the outcome assessor was blinded to the treatment the patients received (Supplementary File S1, Supplementary Figure S3).

### 3.4. Participants

Details about participants, interventions, and outcome measures are reported in Supplementary File S1, Table S1.

The total sample of patients involved in the included studies was 548, resulting from the sum of 461 participants randomized to receive dual-task gait training [9,24,25,26,27,28,29,31,32,33,34,35,36] and 87 participants randomized to receive single-task gait training included in three studies [28,33,36]. These three studies [28,33,36] were specifically chosen for meta-analyses because the comparison group was engaged in single-task gait training rather than passive treatment or no treatment.

The mean age of participants was 66.5 ± 7.2 years for people receiving dual-task gait training and 67.4 ± 8.4 years for people receiving single-task gait training. The sex distribution was comparable between the two groups, and the mean PD duration was 6.7 ± 4.2 years for participants receiving dual-task training and 6.0 ± 5.3 years for people receiving single-task gait training. The mean H&Y score was 2.4 ± 0.5 for participants receiving dual-task gait training and 2.4 ± 0.6 for participants receiving single-task gait training.

### 3.5. Intervention

In eight studies [26,27,30,31,32,33,35,36], the experimental group underwent gait exercises along with concurrent cognitive training. Gait training involved activities such as normal speed forward walking, S-shaped route walking, tandem walking, backward walking, and fast walking. The dual-task included concurrent cognitive activities like walking while repeating words, counting backward a 3-digit number, performing daily cognitive tasks (reciting a shopping list, talking to the phone, opening or closing doors), or engaging in verbal fluency and working memory tasks. In two studies [28,29] dual-task gait training consisted of treadmill training in a virtual reality environment (V-TIME): a camera (Kinect) collected the foot movements of participants while walking (gait training) on the treadmill and incorporated them into computer-generated stimulation, which was presented to participants over a screen placed in front of the treadmill. Patients were required to walk on the treadmill while avoiding virtual obstacles projected on the screen (cognitive training), engaging several cognitive domains such as decision-making and planning, attention (e.g., ignoring distractors on the way), working memory (e.g., navigation), and visual processing (e.g., timing of motor planned action). In three studies [9,24,34], the participants in the experimental group were first subjected to balance training and then to combined walking training with concomitant cognitive tasks. In particular, in two studies [24,34] a specific framework for balance training in pwPD (HiBalance) was used. It consisted of exercises to improve balance performance, gait performance, and strategies of attention in varying balance conditions through increased level of difficulty and task variation for each balance component separately, and by using multi-tasking (i.e., cognitive secondary tasks). Instead, in the third study [9], the patients underwent walking and balance training of increasing difficulty (normal walking, overcoming obstacles, tandem) and in the meantime performed a cognitive task (counting by subtracting “3”, verbal fluency tasks, and pronouncing words backwards). Finally, one study [25] used as an intervention a community-based tango program. Patients were asked to learn new steps and walk while performing cognitive task (naming).

The frequency of training sessions ranged from one [35] to three [9,24,27,28,29,31,34,35] per week, and each session lasted from 30 [36] to 120 min [35]. The mean duration of training was 8.6 ± 2.4 weeks for participants receiving dual-task and 9.3 ± 3.0 weeks for participants receiving single-task gait training.

### 3.6. Outcomes

Thirteen studies assessed spatiotemporal gait parameters [9,24,25,26,27,28,29,31,32,33]. Gait speed was assessed in 11 studies [9,24,25,28,29,31,32,33,34,35,36], stride length was assessed in four studies [26,29,33,36], step length was assessed in three studies [24,31,34], cadence was assessed in five studies [24,26,31,33,36] and dual-task cost on gait speed was assessed in three studies [9,27,36]. Furthermore, three studies [28,30,32] evaluated walking ability by counting the steps that patients took on average in one day. As regards the measuring instruments, spatiotemporal gait parameters were measured through instrumented walkaways (GAITrite, CIR Systems, Inc. (Franklin, NJ, USA), Havertown, Pennsylvania, and Zeno Walkway, ProtoKinetics, Havertown, PA, USA) in six studies [24,25,26,28,32,34,36] and with stereophotogrammetry (BTS GAITLAB, BTS Bioengineering, Garbagnate Milanese, MI, USA, Italy, KINESCAN/IBV photogrammetry, Valencia, Spain, Vicon Gait analysis system, Vicon motion systems, Oxford, UK) in three studies [9,31,33]. Four other studies [27,29,30,35] measured gait parameters through wearable sensors and a recording platform.

Balance confidence during walking was analyzed in four studies [9,29,35,36]. Two of them [29,36] used the Falls Efficacy Scale International (FES-I) as an outcome measure. The other two studies [9,35] assessed balance confidence through the Activity-specific Balance Confidence (ABC) scale.

Finally, the outcome quality of life was analyzed in seven studies [9,24,25,27,28,33,34]. Four of them used the Parkinson’s Disease Questionnaire (PDQ-39) as an outcome measure. This questionnaire assesses how often pwPD experience difficulties across eight dimensions of daily living. Likewise, the difficulties in activities of daily living that reduce the quality of life were measured by three studies [24,25,34] through part II of the UPDRS. The remaining study [28] measured quality of life using the Short Form Health Survey (SF-36).

### 3.7. Efficacy of Dual-Task Training vs. Single-Task Gait Training

#### 3.7.1. Speed

**Figure 1:**
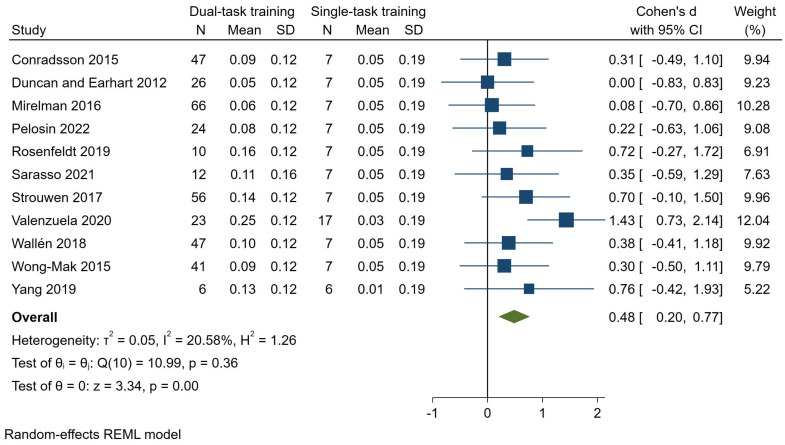
Forest plot for dual-task gait speed. Dual-task gait training vs. single-task gait training in people with PD (data of the control groups taken from Mirelman et al. [28]). Outcome: dual-task gait speed [8,24,25,28,29,31,32,33,34,35,36].

**Figure 2:**
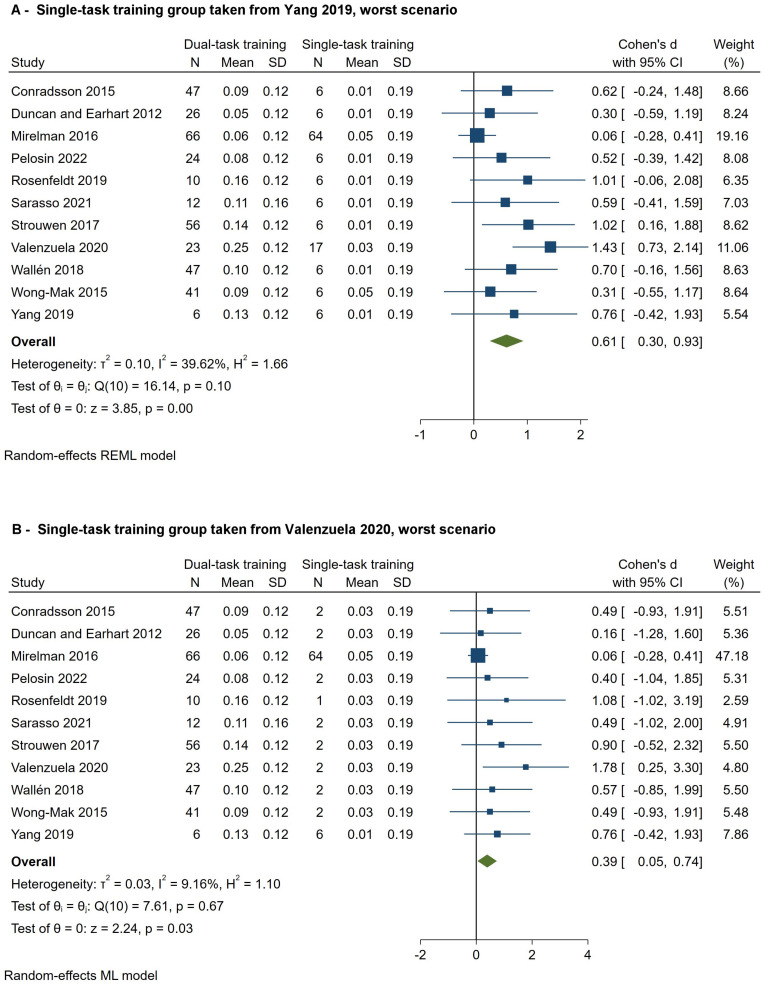
Sensitivity analysis for dual-task gait speed. Sensitivity analysis: dual-task gait training vs. single-task gait training (data of the control groups taken from Yang et al. [36] and Valenzuela et al. [33]). Outcome: dual-task gait speed [8,24,25,28,29,31,32,33,34,35,36].

**Figure 3:**
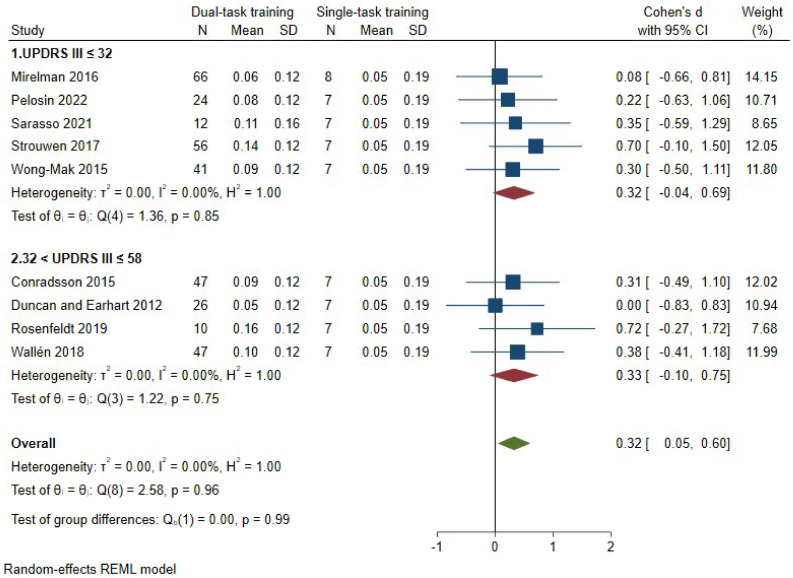
Subgroup analysis for dual-task gait speed. Dual-task gait training vs. single-task gait training in people with PD, subgroups according to the mean baseline UPDRS-III score. Outcome: dual-task gait speed [8,24,25,28,29,31,32,34,35].

Eleven studies [9,24,25,28,29,31,32,33,34,35,36], with a total of 358 patients enrolled in the dual-task training group, assessed dual-task gait speed. The pooled results indicated a significant effect size of 0.48 SMD (95% CI—0.20–0.77, *p* = 0.00, *I*^2^ = 21%) (Figure 1) favoring dual-task gait training over single-task gait training. According to GRADE, we judged the certainty of the evidence to be ‘low’ (Table 1). Sensitivity analyses using data extracted from other studies [33,36], revealed a stable effect of dual-task over single-task gait training (Figure 2). Subgroup analysis according to the UPDRS-III score [23] showed no differences in the estimated effects (*p* = 0.99) (Figure 3).

#### 3.7.2. Stride Length

**Figure 4:**
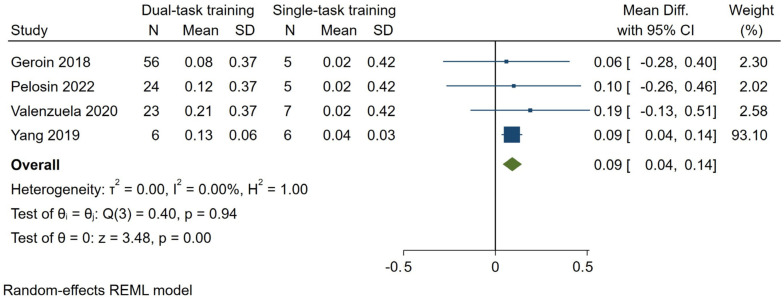
Forest plot for dual-task stride length. Dual-task gait training versus single-task gait training in people with PD. Outcome: dual-task stride length (m) [26,29,33,36].

Four studies [26,29,33,36], with a total of 109 patients randomized to the dual-task gait training group, assessed dual-task stride length. The pooled results indicated a significant effect of 0.09 m (95% CI—0.04–0.14, *p* = 0.00, I^2^ = 0%) (Figure 4) favoring dual-task gait training over single-task gait training. According to GRADE, we judged the certainty of evidence to be ‘very low’ (Table 1). Three other studies [24,31,34], assessed dual-task step length, reporting improvements in the groups that underwent dual-task gait training.

#### 3.7.3. Cadence

**Figure 5:**
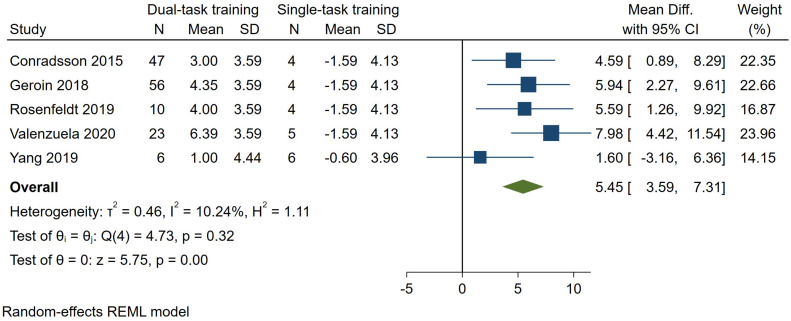
Forest plot for dual-task cadence. Dual-task gait training versus single-task gait training in people with PD. Outcome: dual-task cadence (steps/min) [24,26,31,33,36].

Five studies [24,26,31,33,36], involving 142 patients enrolled in the dual-task gait training groups, assessed dual-task cadence. The pooled results indicated a significant effect of 5.45 steps per minute (95% CI—3.59–7.31, *p* = 0.00, I^2^ = 10%) (Figure 5) favoring dual-task gait training over single-task gait training. Using GRADE, we judged the certainty of evidence to be ‘very low’ (Table 1).

**Table 1:** GRADE, primary outcomes.

Certainty Assessment							№ of Patients		Effect		Certainty	Comments
№ of Studies	Study Design	Risk of Bias	Inconsistency	Indirectness	Imprecision	Other Considerations	Dual-Task Gait Training	Single-Task Gait Training	Relative (95% CI)	Absolute (95% CI)		
Dual-task speed (assessed with: m/s)												
11	randomized trials	not serious	not serious	very serious (a)	not serious	none	358	86	-	0.48 standard deviations higher (0.20 to 0.77 higher)	⨁⨁◯◯ Low	Moderate effect favoring dual-task gait training relative to single-task gait training in improving dual-task speed in people with PD
Dual-task stride length (assessed with: m)												
4	randomized trials	serious (b)	not serious	very serious (c)	not serious	none	109	23	-	0.09 m higher (0.04 to 0.14 higher)	⨁◯◯◯ Very Low	Dual-task relative to single-task gait training leads to higher dual-task stride length improvement (0.09 m) in people with PD
Dual-task cadence (assessed with: steps/min)												
5	randomized trials	serious (d)	not serious	very serious (c)	not serious	none	142	23	-	5.45 steps/min higher (3.59 to 7.31 higher)	⨁◯◯◯ Very Low	Dual-task relative to single-task gait training leads to higher dual-task cadence improvement (5.62 steps/min) in people with PD

Abbreviations: CI—confidence interval; m—meters. Explanations: a. One trial included in the meta-analysis evaluated turning speed rather than straight gait speed. Furthermore, one comparison group was used for the other studies. b. 50% of trials are considered to have a “high” or “some concerns” risk of bias for this outcome. c. One comparison group was used for the other studies. d. More than 50% of trials are considered to have a “high” or “some concern risk of bias for this outcome.

#### 3.7.4. Secondary Outcomes

We found a significant effect of dual-task gait training over single-task gait training on dual-task cost on gait speed and quality of life, not on balance confidence. Three studies [9,27,36] involving 44 patients in the dual-task gait training group evaluated the outcome of dual-task cost on gait speed by expressing performance changes under the dual-task condition as a percentage of each individual’s single-task performance on the respective task. The pooled results indicated a significant effect size of 0.99 SMD (95% CI—0.10–1.89, *p* = 0.03, I^2^ = 0%) (Supplementary File S1, Supplementary Figure S4) favoring dual-task over single-task gait training. Using GRADE, we judged the certainty of the evidence to be ‘very low’ (Supplementary File S1, Table S2). Four studies [9,29,35,36] involving 84 patients in the dual-task gait training group evaluated the outcome of balance confidence. Two of these [29,36] used the Falls Efficacy Scale (FES-I) as an outcome measure. The other two [9,35] used the Activity-specific Balance Confidence (ABC) scale. The pooled results indicated a non-significant effect size of 0.63 SMD (95% CI—0.27–1.53, *p* = 0.17, I^2^ = 0%) (Supplementary File S1, Supplementary Figure S5) favoring dual-task over single-task gait training. Using GRADE, we judged the certainty of the evidence to be ‘very low’ (Supplementary File S1, Table S2). Finally, seven studies [9,24,25,27,28,33,34] that included 247 subjects in dual-task training analyzed the outcome quality of life. Four studies [9,25,27,33] assessed the outcome using the PDQ-39, one study [28] used the Short Form Health Survey 36 (SF-36), and two studies [24,34] used the UPDRS-II. The pooled results indicated a significant effect size of 0.55 SMD (95% CI—0.02–1.08, *p* = 0.04, I^2^ = 74%) (Supplementary File S1, Supplementary Figure S6) favoring dual-task gait training over single-task gait training. Using GRADE, we judged the certainty of the evidence to be ‘very low’ (Supplementary File S1, Table S2).

## 4. Discussion

The aim of this study was to assess the effect of dual-task training relative to single-task training of gait on specific gait parameters during dual-task tests in pwPD and to investigate the possible effect modifiers. We included and pooled data from 13 trials and found evidence of some beneficial effects of dual-task gait training over single-task gait training on dual-task gait parameters, dual-task cost, and quality of life.

Data show an improvement in dual-task gait speed, stride length, and cadence, which are usually affected in pwPD, particularly during attention-demanding tasks [37], and tend to worsen with disease progression [38]. As expected, improvements in gait were present even in patients undergoing single-task gait training. Indeed, it is well known that gait training has a positive effect on spatiotemporal gait parameters [39]. However, dual-task gait training promotes an additional modest benefit over single-task gait training on dual-task gait speed (0.48 SMD). The estimated effect is clinically relevant considering that dual-task walking speed increased by 0.10 m/s after dual-task training relative to single-task training of gait in a population of pwPD with impaired walking speed (ranging from 0.92 to 1.3 m/s) [40]. Furthermore, this finding is particularly relevant considering that a lower gait speed is associated with a higher rate of falls, cognitive impairment, and functional decline in the elderly and pwPD [41,42,43,44]. Sensitivity analyses confirmed the robustness of our estimation regarding the superiority of dual-task over single-task training of gait. However, caution should be exercised when interpreting the overall results of the meta-analysis on dual-task gait speed.

Moreover, we performed a sub-analysis dividing subjects based on the UPDRS-III score at study entry to assess if the effect on dual-task gait speed was different according to disease severity. No differences were found comparing studies enrolling people with greater motor impairment (32 < UPDRS-III ≤ 58) (0.32 SMD, 95% CI—−0.05–0.60, *p* = 0.75, I^2^ = 0%) relative to those enrolling people with less motor impairment (UPDRS-III ≤ 32) (0.32 SMD, 95% CI—0.04–0.69, *p* = 0.85, I^2^ = 0%) (test for subgroup differences, *p* = 0.99).

Meta-analyses results showed that dual-task gait training relative to single-task gait training has a greater effect on dual-task stride length (MD 0.09 m) with a ‘very low’ certainty of evidence. This finding, taken together with the gait speed improvement, has a significant clinical implication for walking safety. In fact, an increased dual-task gait speed associated with a higher dual-task stride length might reflect more stable and safe walking and, consequently, a reduced risk of falls [45]. The improvement of dual-task cost on gait speed (0.99 SMD) further corroborates this hypothesis, suggesting that dual-task gait training can reduce dual-task interference and ameliorate gait automaticity.

Also analyzing the dual-task cadence outcome measure, we found a larger improvement in patients undergoing dual-task gait training compared to single-task gait training (MD 5.45 steps/min) with a very low certainty of evidence. The increased step cadence is associated with a higher walking speed and stride length, thus suggesting more efficient walking and a low probability of a shuffling gait or a freezing of gait manifestation [46].

Dual-task gait training seems not to be more useful compared to single-task gait training in improving balance confidence (0.63 SMD with a ‘very low’ certainty of evidence). This finding is surprising as dual-task training consists of complex exercises that engage multiple systems simultaneously, making it challenging for balance maintenance in pwPD. Indeed, pwPD usually need a high level of cognitive control over movement to avoid imbalance [47,48]. Dual-task training requires a shift of attention from the movement to the cognitive task, thus promoting gait automaticity [49]. All these aspects should support the enhancement of motor automaticity, which could potentially improve perceptions of motor control and safety while walking. However, the limited number of studies evaluating balance confidence, along with gaps in data estimation, lead us to estimate a non-significant effect of dual-task training compared to single-task training of gait. Confidence in this finding is very low, indicating poor reliability of the available effect estimates. Moreover, new studies have the potential to entirely alter the estimated effect.

Pooling data from the included studies also indicates an enhancement in the quality of life for pwPD undergoing dual-task gait training compared to those undergoing single-task gait training (0.55 SMD with a very low certainty of evidence). One plausible explanation is that improvements in walking and other gait parameters may have positively influenced the quality of life of pwPD, thereby suggesting the clinical relevance of the beneficial outcomes of dual-task training.

The overall risk of bias in the included studies, according to the RoB 2 assessment, is high, mainly due to incomplete information on the randomization process and allocation concealment and a lack of pre-published study protocols. Sensitivity analysis for the outcome dual-task gait speed according to the overall risk of bias revealed a non-significant effect of dual-task gait training on gait speed (0.33 SMD, 95% CI—−0.04–0.70, *p* = 0.86, I^2^ = 0%) (Supplementary File S1, Supplementary Figure S7).

Our results are in line with those of other recently published reviews, suggesting that dual-task gait training is useful to improve dual-task gait parameters such as gait speed, step length, and cadence in PD subjects [13,14,15,50]. As previously suggested [15], we also found a significant effect on dual-task cost. Other reviews investigated other domains such as balance [13,50] and cognitive functions [14], suggesting a further effect of dual-task gait training on these abilities. However, previous reviews included control groups regardless of the type of training proposed (e.g., usual care, walking training alone, no treatment). To the best of our knowledge, this is the first review using only well-defined single-task gait training as a comparator, and no other active or passive control groups to identify what a dual-task training of gait could specifically add to a “gold standard” single-task training of gait. Moreover, this is the only review that stratifies patients according to their motor abilities at baseline, suggesting that dual-task gait training can be administered with consistent results both in mild and moderate pwPD [23].

### Limitations

Our review aimed to assess the effectiveness of dual-task gait training compared to an active treatment, specifically single-task gait training, to better gauge its impact on dual-task gait speed. Our results are in line with the recent review by Wong et al., which examined the impact of dual-task gait training compared to various comparisons [14]. Specifically, they identified ten trials investigating dual-task training’s effects on dual-task walking performance, with half of these trials contrasting dual-task gait training with non-active interventions. In consideration of the paucity of studies addressing our primary aim, we want to draw the readers’ attention to specific aspects clarifying our methodological choices in conducting this review, which should be considered essential for interpreting and discussing the possible strengths and limitations of our findings.

Firstly, in order to retain insights from studies comparing dual-task training with other intervention types, in comparing dual-task training to single-task training of gait, our synthesis and analyses are grounded in the effects of treatments exclusively applied in studies using single-task gait training as a comparison [28,33,36]. In fact, other included studies compared dual-task gait training to consecutive training of motor and cognitive tasks [26,30,31,32], other active comparisons [9,29,35], or no intervention [24,25,27,34]. The inclusion of comparison groups from a limited pool of studies may potentially impact the precision of our estimates. However, we tried to address the unit-of-analysis error by dividing the total number of participants in the selected single-task gait training group by the number of comparisons. We acknowledge the potential for this approach to yield a misleading response to our primary inquiry. Therefore, we sought to validate the robustness of our findings by conducting sensitivity analyses consisting of identical analyses using data from two comparator groups sourced from other studies. The sensitivity analysis consistently demonstrated a stable effect, regardless of the chosen comparator group. However, even if sensitivity analyses support the robustness of our estimation of the efficacy of dual-task training compared to single-task training of gait, caution should be used in interpreting the results on dual-task gait speed. We considered these aspects in determining the certainty of evidence, by lowering the strength of evidence by two levels in the GRADE framework. Through exploratory analyses not reported in this review, we investigated the effects of dual-task versus single-task gait training, focusing on post-treatment data from three studies enrolling 182 participants and directly comparing these interventions [28,33,36]. These analyses revealed a statistically significant and clinically relevant effect favoring dual-task gait training in improving dual-task gait speed with a significant effect size of 1.4 SMD (95% CI—0.89–1.91, *p* = 0.00, I^2^ = 42%). Our findings, incorporating dual-task gait training interventions compared with the single-task gait training group from the study of Mirelman et al. [28], which exhibits the lowest risk of bias and participant similarity to those in dual-task gait training studies, align with these preliminary assessments. Furthermore, this review aimed to ascertain the unique contribution of dual-task gait training over single-task gait training, and we examined the post- and pretreatment differences between both groups. This approach helped alleviate baseline participant variations and further minimize the influence of contrasting groups from disparate studies. Considering these factors, we deemed it appropriate to utilize a single comparator across multiple studies, with the sole purpose of retaining insights from trials employing dual-task gait training and enhancing specificity in determining its effects compared to single-task gait training.

Secondly, we had to face moderate clinical heterogeneity among the included trials regarding proposed interventions and the clinical characteristics of participants. Two studies [28,29] utilized a treadmill, while others employed overground walking to train in the dual-task condition. Due to evidence suggesting non-inferiority between these methods [51], we combined and analyzed the data. With the currently available data, patient stratification according to crucial clinical characteristics such as the degree of cognitive impairment, the presence of freezing of gait, or falls is challenging, as only a few studies have provided a thorough screening of these clinical features at baseline. To address this clinical heterogeneity, we utilized random-effects models for analyses, adopting a conservative approach in our estimation to consider both clinical and statistical heterogeneity.

Lastly, the included studies demonstrated heterogeneity in outcome determination during dual-task gait assessments. The administered cognitive tasks assessed various cognitive domains, with the majority using executive tasks but with different requests (e.g., counting backward, verbal fluency, or Stroop test). Furthermore, we were unable to assess the effects of dual-task training on falls. It is quite unexpected that only four studies [28,29,30,32] assessed this outcome. Indeed, it has great relevance and potential impact on quality of life, hospitalization, and possible economic consequences. Moreover, the four studies that assessed falls used different outcome measures (incident rate of falls, frequency of falls, number of falls) and reported over different time points (1-month, 6-month), thus providing incomparable information.

## 5. Conclusions

In conclusion, our findings support the use of dual-task training relative to single-task training of gait to improve dual-task spatiotemporal gait parameters in pwPD. However, we recommend that future studies improve the reporting according to the CONSORT guidelines [52], particularly concerning the training description and clinical characteristics of the included participants. This would help to ensure the transparency and reproducibility of research, allowing readers to critically evaluate the content of interventions and identify those subjects who might benefit more from dual-task gait training. Additionally, for future research, it would also be necessary to define a homogeneous core outcome set to assess dual-task gait in pwPD.

## Data Availability

The datasets generated during and/or analyzed during the current study are available from the corresponding author upon reasonable request.

## Supplementary Materials

The following supporting information can be downloaded at: https://www.mdpi.com/article/10.3390/brainsci14050517/s1, File S1: Search strategies, Flow of the screening process; Figure S1: PRISMA flow diagram; Figure S2: Risk of bias; Figure S3: Traffic light plot; Figure S4: Forest plot for dual-task cost on gait speed; Figure S5: Forest plot for balance confidence; Figure S6: Forest plot for quality of life; Figure S7: Sensitivity analysis according risk of bias. Outcome: dual-task gait speed. Table S1: Main characteristics and outcomes of the included studies; Table S2: GRADE, secondary outcomes. References [53,54,55,56,57,58,59,60,61,62,63,64,65,66,67,68,69,70,71,72,73,74,75,76,77,78,79,80,81] are cited in Supplementary Materials.

## Abbreviations

PD	Parkinson Disease
pwPD	people with Parkinson Disease
PRISMA	Preferred Reporting Items for Systematic Reviews and Meta-Analyses
CENTRAL	Cochrane Central Register of Controlled Trials
RCTs	Randomized controlled trials
UPDRS	Unified Parkinson’s Disease Rating Scale
H&Y	Hoehn and Yahr
RoB 2	Version 2 of the Cochrane risk-of-bias tool for randomized trials
SDs	Standard Deviations
MD	Mean Difference
SMD	Standardized Mean Difference
CI	Confidence Interval
GRADE	Grading of Recommendation Assessment, Development and Evaluation
FES-I	Falls Efficacy Scale International
ABC	Activity-specific Balance Confidence Scale
PDQ-39	Parkinson’s Disease Questionnaire
SF-36	Short Form Health survey
