# Supplementary material for: Dual-Task vs. Single-Task Gait Training to Improve Spatiotemporal Gait Parameters in People with Parkinson’s Disease: A Systematic Review and Meta-Analysis
Source: Brain Sci. 2024 May 20;14(5):517. doi: 10.3390/brainsci14050517 (PMC11119953; doi:10.3390/brainsci14050517)
Supplement: Supplementary file 1 [file brainsci-14-00517-s001.zip › brainsci-2985548-supplementary.pdf]

Supplementary file S1

Search strategies

We used different search strategies for each of the three databases: Pubmed, Embase, CENTRAL.

Pubmed

(((Parkinson*[TW] OR "Parkinson disease"[Mesh] OR "paralysis agitans"[TW]) AND ("Walking"[Mesh] OR "Gait"[Mesh] OR (walk*[TIAB] OR gait[TIAB] OR ambulat*[TIAB] OR mobil*[TIAB] OR locomot*[TIAB] OR stride*[TIAB] OR stroll*[TIAB] OR treadmill*[TIAB]) OR (walk* [TW] OR gait[TW] OR ambulat*[TW] OR mobil*[TW] OR locomot*[TW] OR stride*[TW] OR stroll*[TW] OR treadmill*[TW])) AND (dualtask[TW] OR dual-task[TW] OR dualtasking[TW] OR dual-tasking[TW] OR multimodal[TW] OR multi-modal[TW] OR multi-dimensional[TW] OR multidimensional[TW])) AND ("randomized controlled trial"[PT] OR "controlled clinical trial"[PT] OR "randomized"[TIAB] OR "randomised"[TIAB] OR "placebo"[TIAB] OR "clinical trials as topic"[Mesh] OR "randomly"[TIAB] OR "trial"[TI])) NOT ("animals"[Mesh] NOT "humans"[Mesh])

Embase

#1 (parkinson*:ti,ab OR 'parkinson disease'/exp OR 'parkinson disease' OR 'paralysis agitans':ti,ab) AND ('gait'/exp OR 'gait' OR 'walking'/exp OR 'walking' OR 'gait speed':ti,ab OR 'walking speed'/exp OR 'walking speed' OR 'walking parameters'/exp OR 'walking parameters' OR 'walking difficulty'/exp OR 'walking difficulty' OR 'falling'/exp OR 'falling' OR 'fall risk'/exp OR 'fall risk' OR 'fall risk assessment'/exp OR 'fall risk assessment' OR 'mobilization'/exp OR 'mobilization' OR 'locomotion'/exp OR 'locomotion') AND ('dualtask' OR 'dual-task' OR

'dualtasking' OR 'dual-tasking' OR 'multimodal' OR 'multi-modal' OR 'multi-dimensional' OR 'multidimensional') AND ('randomized controlled trial'/exp OR 'randomized controlled trial' OR 'crossover procedure'/exp OR 'crossover procedure' OR 'placebo'/exp OR 'placebo' OR 'randomization'/exp OR 'randomization' OR 'controlled clinical trial'/exp OR 'controlled clinical trial' OR 'double blind procedure'/exp OR 'double blind procedure' OR 'single blind procedure'/exp OR 'single blind procedure')

#2 [embase]/lim OR [preprint]/lim

#3 #1 AND #2

CENTRAL

MeSH descriptor: [Parkinson Disease] explode all trees

#2 (Parkinson*):ti,ab,kw

#3 ("paralysis agitans"):ti,ab,kw

#4 #1 or #2 or #3

#5 MeSH descriptor: [Walking] explode all trees

#6 MeSH descriptor: [Gait] explode all trees

#7 (gait):ti,ab,kw OR (ambulat*):ti,ab,kw OR (mobil*):ti,ab,kw

#8 (locomot*):ti,ab,kw OR (stride*):ti,ab,kw OR (stroll*):ti,ab,kw OR (treadmill*):ti,ab,kw

#9 #5 or #6 or #7 or #8

#10 (dualtask):ti,ab,kw OR (dualtasking):ti,ab,kw OR (dual-task):ti,ab,kw OR (dual-tasking):ti,ab,kw

#11 (multimodal):ti,ab,kw OR (multi-modal):ti,ab,kw OR (multidimensional):ti,ab,kw OR (multi-dimensional):ti,ab,kw

#12 #10 or #11

#13 ("randomized controlled trial"):pt OR ("controlled clinical trial"):pt OR ("randomized"):ti,ab OR ("randomised"):ti,ab OR ("placebo"):ti,ab

#14 ("randomly"):ti,ab OR (“clinical-trial”):ti,ab OR ("trial"):ti

#15 MeSH descriptor: [Clinical Trials as Topic] explode all trees

#16 #13 or #14 or #15

#17 (animals) not (humans)

#18 #16 not #17

#16 #4 and #9 and #12 and #18

Flow of the screening process – exclusion criteria

Two papers were excluded because they were non-randomized trials (1, 2) . Ten more papers were excluded because there was not an administration of a dual-task gait intervention (3-12) and 10 were exclude because they were single-session studies (13-22). Moreover, eight (23-30) studies were excluded because no dual-task gait parameters data useful for the analysis were reported in the article.

References of excluded studies

1. Mirelman A, Maidan I, Herman T, Deutsch J, Giladi N, Hausdorff J. Virtual reality for gait training: can it induce motor learning to enhance complex walking and reduce fall risk in patients with Parkinson's disease? 2011;66(2):234-40.

2. Nieuwboer A, Munks L, Strouwen C, Molenaars E, Munneke M, Keus S, Rochester L. Dual tasking in Parkinson's disease increases walking speed: the influence of repeated measures. 2012;27:S280.

3. Li Z, Wang T, Shen M, Song T, He J, Guo W, et al. Comparison of Wuqinxi Qigong with Stretching on Single-and Dual-Task Gait, Motor Symptoms and Quality of Life in Parkinson's Disease: A Preliminary Randomized Control Study. Int J Environ Res Public Health. 2022;19(13).

4. Hulbert S, Chivers-Seymour K, Summers R, Lamb S, Goodwin V, Rochester L, et al. 'PDSAFE' - a multi-dimensional model of falls-rehabilitation for people with Parkinson's. A mixed methods analysis of therapists' delivery and experience. Physiotherapy. 2021;110:77-84.

5. Capato T, De Vries N, IntHout J, Ramjith J, Barbosa E, Nonnekes J, Bloem B. A randomized clinical trial of multimodal balance training with rhythmical cues: Effects on freezing of gait in Parkinson's disease. Mov Disord. 2019;34:S32.

6. Beck EN, Intzandt BN, Almeida QJ. Can Dual Task Walking Improve in Parkinson's Disease After External Focus of Attention Exercise? A Single Blind Randomized Controlled Trial. Neurorehabil Neural Repair. 2018;32(1):18-33.

7. Vergara-Diaz G, Osypiuk K, Hausdorff JM, Bonato P, Gow BJ, Miranda JGV, et al. Tai chi for reducing dual-task gait variability, a potential mediator of fall risk in parkinson’s disease: A pilot randomized controlled trial. Global Adv Health Med. 2018;7.

8. Chomiak T, Watts A, Meyer N, Pereira FV, Hu B. A training approach to improve stepping automaticity while dual-tasking in Parkinson's disease. Medicine. 2017;96(5).

9. Brown L, de Bruin N, Doan J, Suchowersky O, Hu B. Novel challenges to gait in Parkinson's disease: the effect of concurrent music in single- and dual-task contexts. 2009;90(9):1578-83.

10. Pohl P, Wressle E, Lundin F, Enthoven P, Dizdar N. Group-based music intervention in Parkinson's disease - findings from a mixed-methods study. Clin Rehabil. 2020;34(4):533-44.

11. Sousa AVC, Simão CR, De Melo Santiago LM, Spaniol AP, Oliveira D, Lindquist RR. Effects of treadmill training on dual-task gait in people with parkinson's disease. Arch Phys Med Rehabil. 2013;94(10):e14-e5.

12. Vieira-Yano B, Martini D, Horak F, de Lima-Pardini A, Almeida F, Santana V, et al. The Adapted Resistance Training with Instability Randomized Controlled Trial for Gait Automaticity. Movement disorders : official journal of the Movement Disorder Society. 2021;36(1):152-63.

13. Silva RDN, Afonso SV, Felipe LR, Oliveira RA, Patrizzi Martins LJ, Pascucci Sande de Souza LA. Dual-task intervention based on trail making test: Effects on Parkinson's disease. J Bodywork Mov Ther. 2021;27:628-33.

14. O’Connell E, Guidon M. Fear of falling and dual-task performance in people with Parkinson’s disease. Europ J Physiother. 2016;18(3):167-72.

15. Sousa AVC, Santiago LMM, Silva REO, Spaniol AP, Oliveira DA, Galvão ÉRVP, et al. Can treadmill training facilitate the dual-task gait in Parkinson's disease? Mov Disord. 2016;31:S649.

16. Pompeu S, Okamoto E, Piemonte ME. Dual-task performance assessment: Motor performance of gait, balance, posture and manual skill in dual-tasks. Physiotherapy. 2011;97:eS1014.

17. Piemonte M, Pikel M, Mendes F, Maciel L, Lopes A. Improvement of the gait performance under dual-task condition after mental practice in patients with Parkinson's disease: a single-blind, randomised clinical trial. 2015;30:S111.

18. Pimentel Piemonte M, Mendes F, Pompeu J, Lobo A, Silva K, Oliveira T, Petersson A. Improvement of gait, functional and cognitive performance in patients with parkinson's disease after motor and cognitive training. 2011;97:eS1002.

19. Criminger C, Swank C. Influence of tDCS on Dual-Task Mobility Elements in Individuals with Parkinson's Disease Using 2D Kinematic Analysis Technology. 2020;101(12):e143.

20. Hu B, De Bruin N, Doan J, Turnbull G, Suchowersky O, Bonfield S, Brown L. Walking with music is a safe and viable tool for gait training in parkinson's disease: the effect of a 13-week feasibility study on single and dual task walking. 2010;2010:Article no: 483530.

21. Bedeschi Ferrari C, Rodrigues L, Bauer D, Manfredi A, Pimentel Piemonte M. Gait training associated with executive functions tasks in subjects with Parkinson's disease: improvement of performance and effects in motor learning. 2012;27:S12.

22. Bedeschi Ferrari C, Rodrigues L, Bauer D, Piemonte M. Improvement of gait, functional and cognitive performance in patients with parkinson's disease after gait training associated with executive function tasks. 2011;97:eS998-eS9.

23. Gaßner H, Trutt E, Seifferth S, Friedrich J, Zucker D, Salhani Z, et al. Treadmill training and physiotherapy similarly improve dual task gait performance: a randomized-controlled trial in Parkinson's disease. J Neural Transm (Vienna). 2022;129(9):1189-200.

24. Silva AZD, Israel VL. Effects of dual-task aquatic exercises on functional mobility, balance and gait of individuals with Parkinson's disease: A randomized clinical trial with a 3-month follow-up. Complement Ther Med. 2019;42:119-24.

25. King L, Smulders K, Mancini M, Lapidus J, Carlson-Kuhta P, Fling B, et al. A combined cognitive and motor exercise program for people with Parkinson's disease and Freezing of gait; a pilot study. Mov Disord. 2017;32:873.

26. Lofgren N, Conradsson D, Rennie L, Moe-Nilssen R, Franzén E. Highly challenging gait and balance training can improve cognitive processing during dual-task conditions in elderly with Parkinson's disease. J Parkinson's Dis. 2016;6:212-3.

27. Kim H, Kim E, Yun SJ, Kang M-G, Shin HI, Oh B-M, Seo HG. Robot-assisted gait training with auditory and visual cues in Parkinson's disease: A randomized controlled trial. Ann Phys Rehabil Med. 2022;65(3).

28. Jabre MG, Elias N, Karam R, Haddad I, Habib K, Bejjani BP. Efficacy of double-task training on gait performance in Parkinson's disease: A randomized, controlled, double-blind study. Mov Disord. 2012;27:S305.

29. Rios Romenets S, Anang J, Fereshtehnejad S, Pelletier A, Postuma R. Tango for treatment of motor and non-motor manifestations in Parkinson's disease: a randomized control study. Complementary therapies in medicine. 2015;23(2):175-84.

30. Vitório R, Teixeira-Arroyo C, Lirani-Silva E, Barbieri F, Caetano M, Gobbi S, et al. Effects of 6-month, Multimodal Exercise Program on Clinical and Gait Parameters of Patients with Idiopathic Parkinson's Disease: A Pilot Study. ISRN neurology. 2011;2011:714947.

Supplementary figures

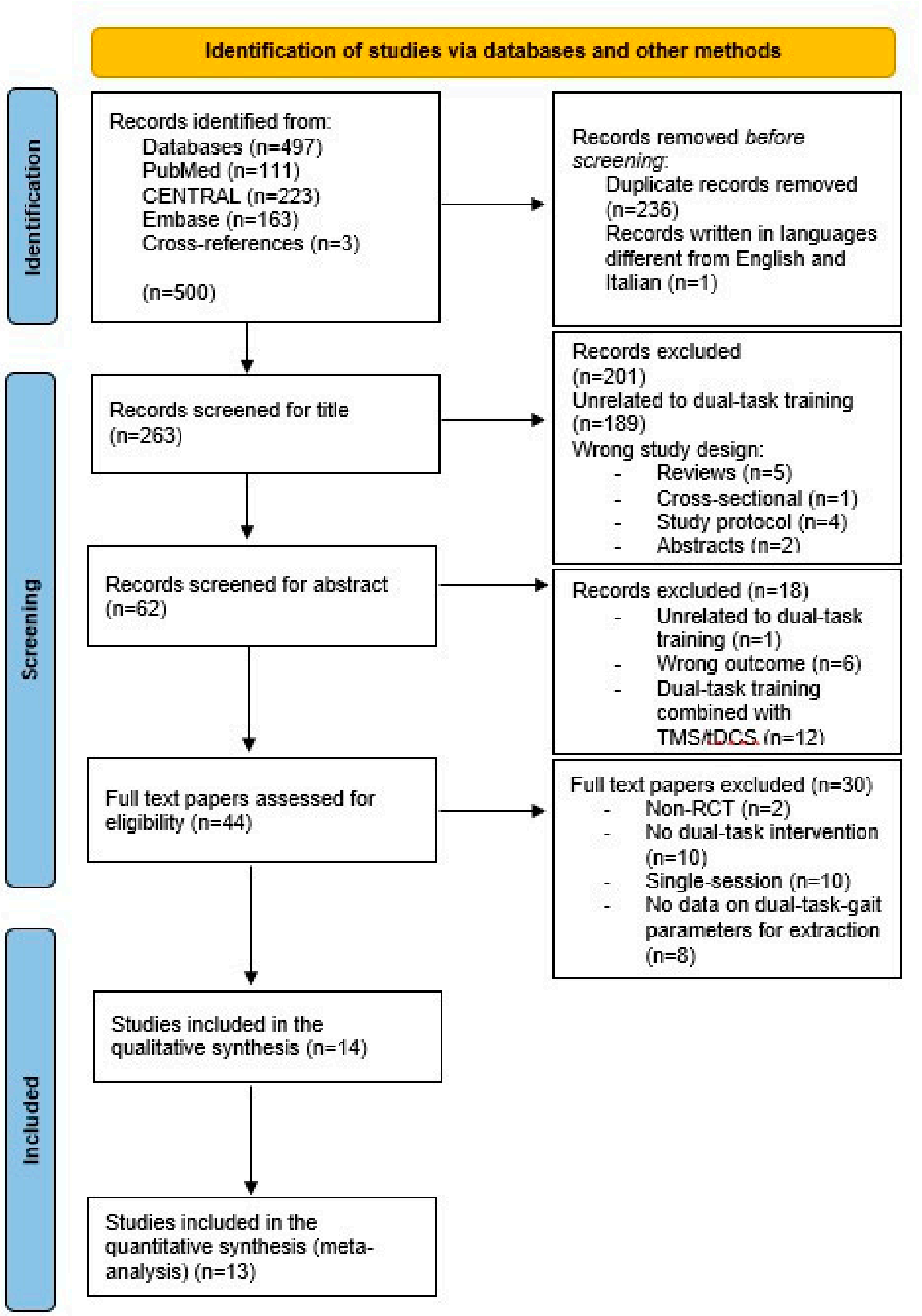

Supplementary Figure S1 - PRISMA flow diagram

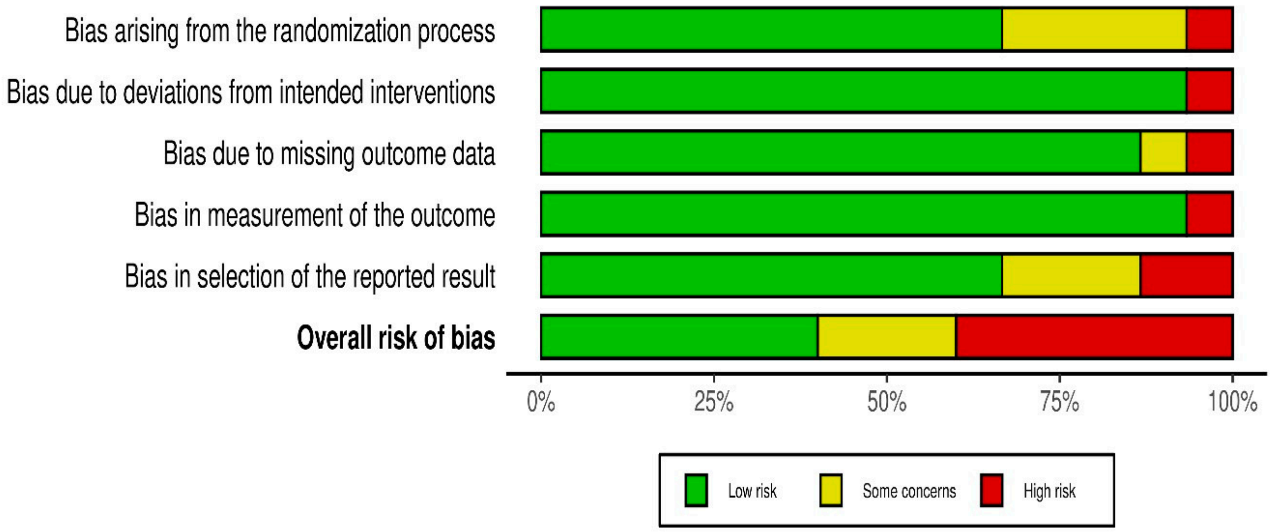

Supplementary Figure S2 - Risk of bias

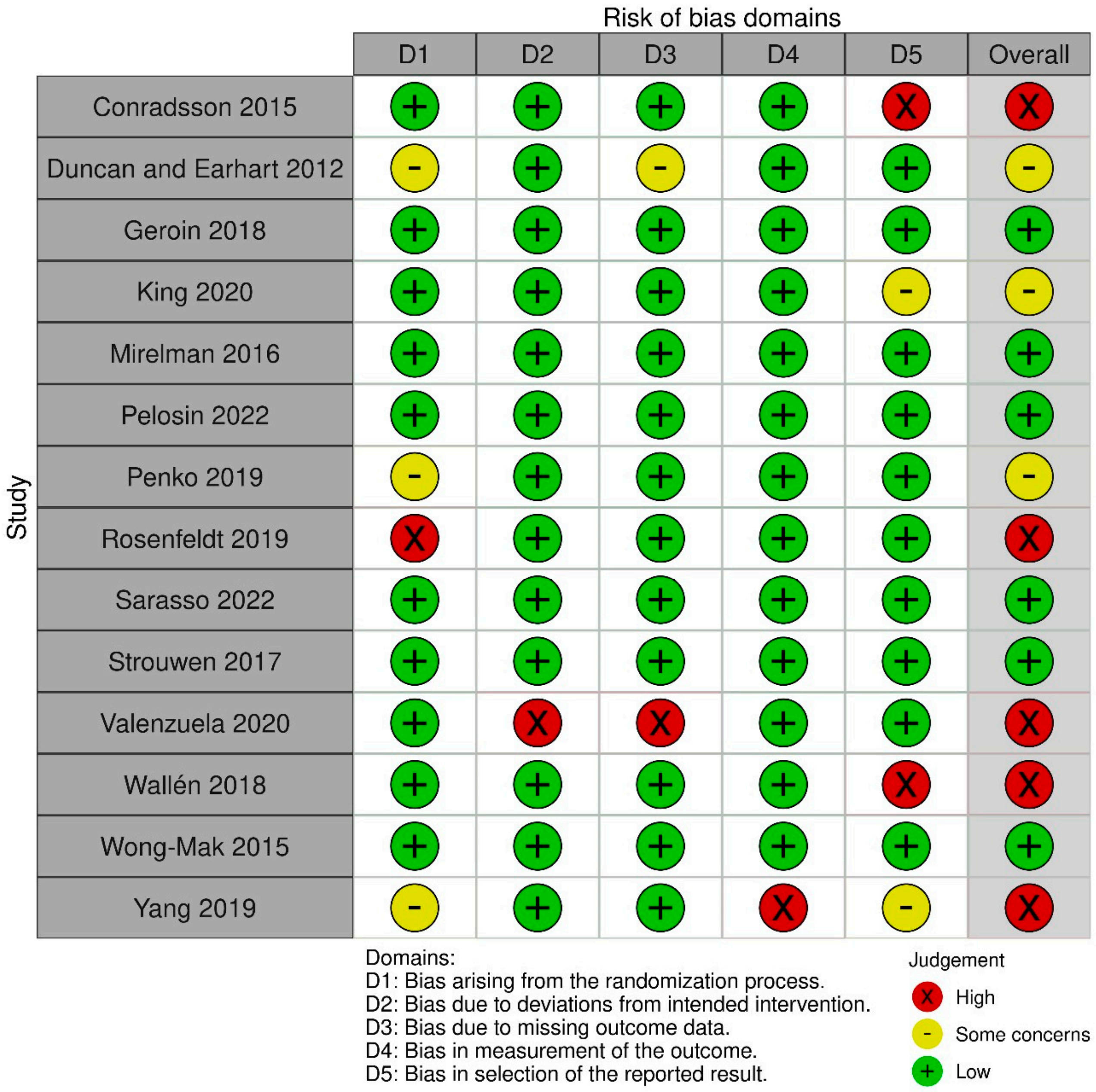

Supplementary Figure S3 - Traffic light plot

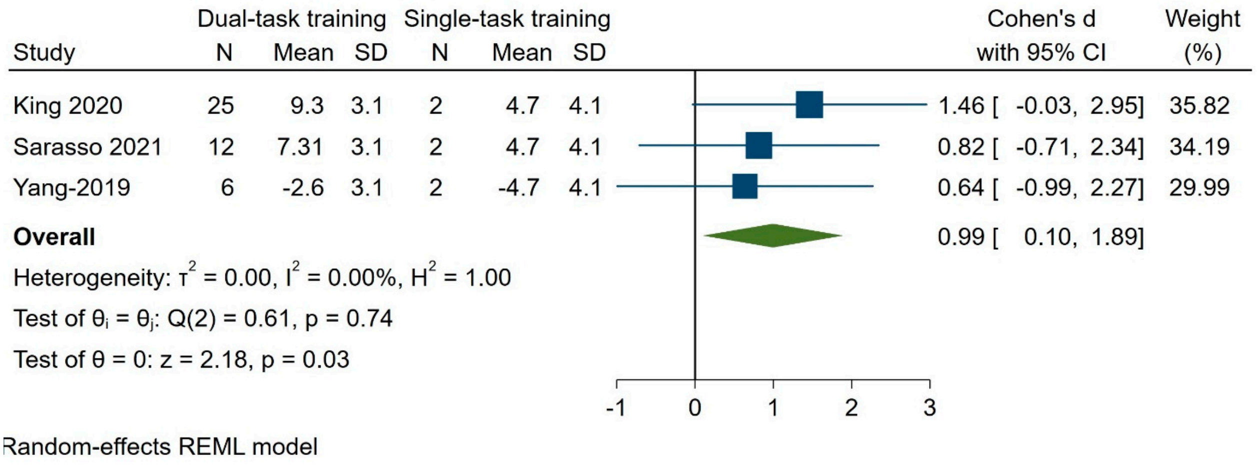

Supplementary Figure S4 - Forest plot for dual-task cost on gait speed

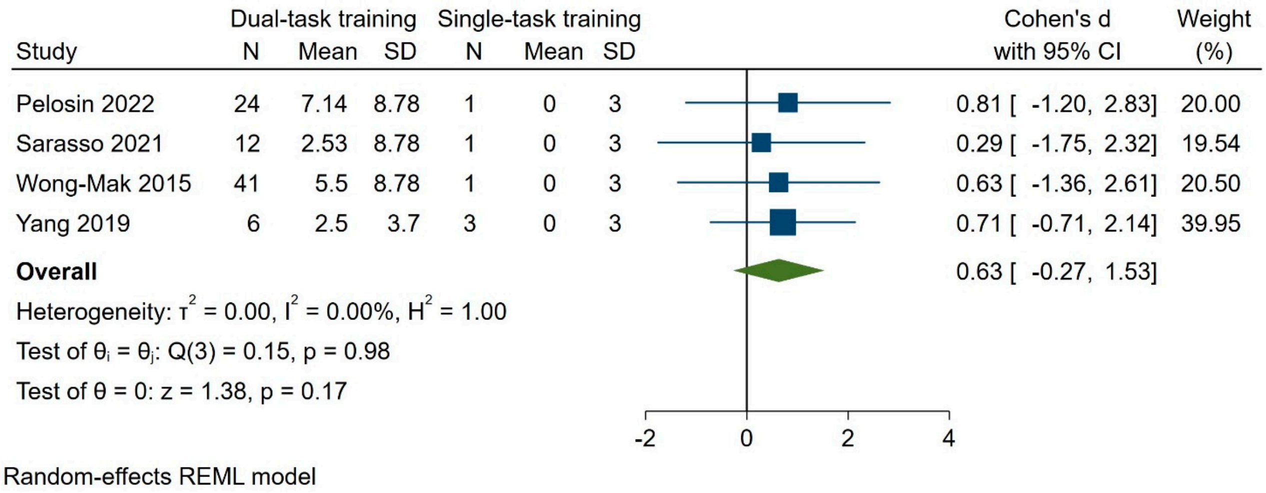

Supplementary Figure S5 - Forest plot for balance confidence

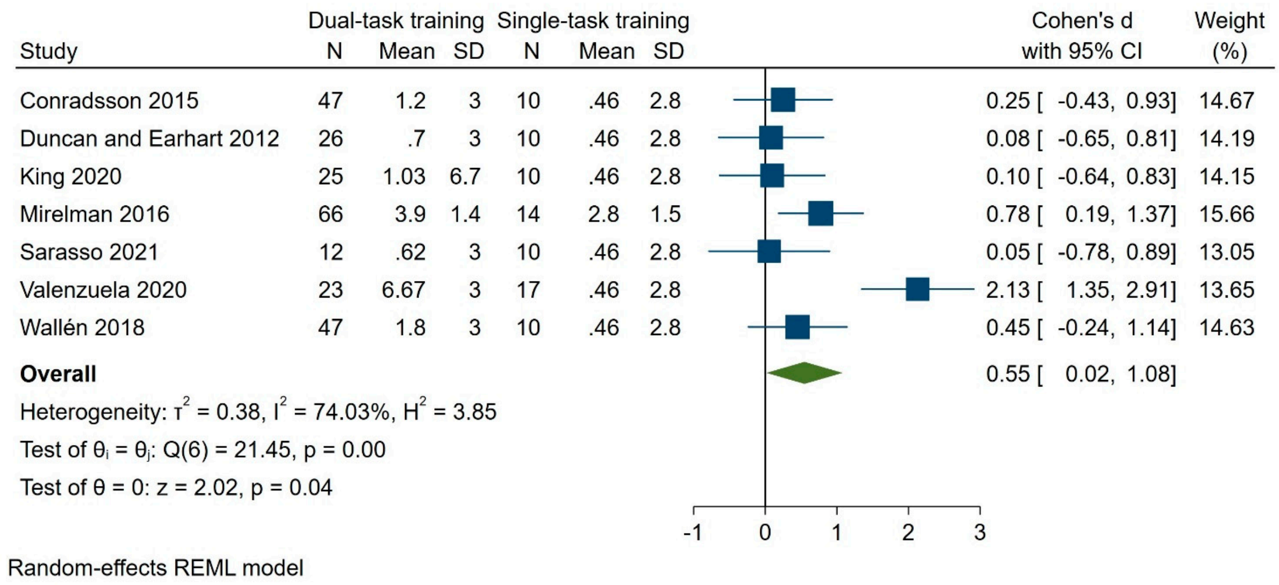

Supplementary Figure S6 - Forest plot for quality of life

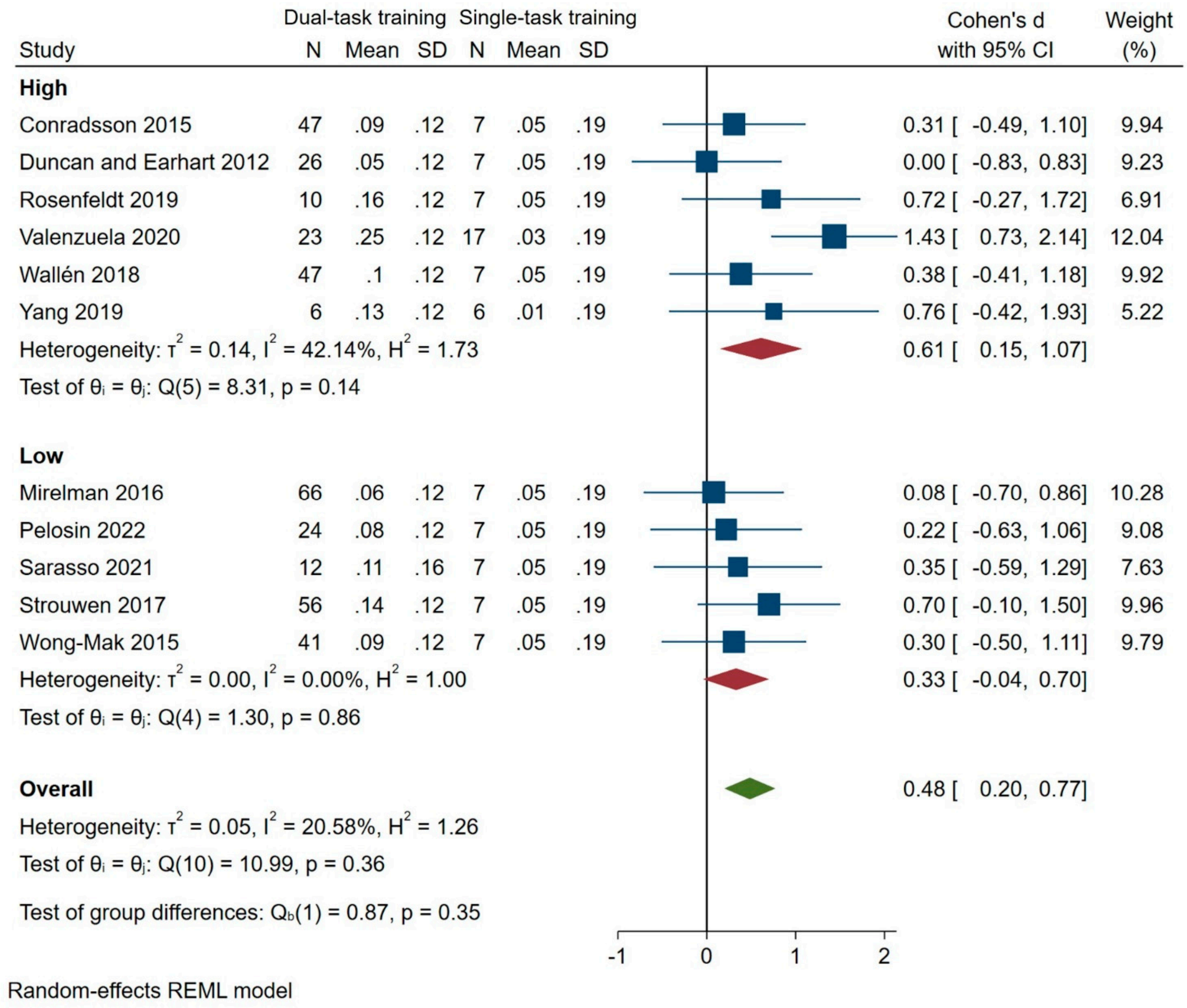

Supplementary Figure S7 - Sensitivity analysis according risk of bias. Outcome: dual-task gait speed.

Table S1. Main characteristics and outcomes of the included studies.

AUTHOR	COUNTRY	SAMPLE	H&Y	UPDRS-III AT BASELINE	TREATMENT	ON/OFF	CONTROL	DURATION (WEEKS)	FREQUENCY (WEEKLY SESSIONS X MINUTES)	GAIT ANALYSIS	GAIT OUTCOME
**Conradsson 2015**	Sweden	47	EX 2.6 (0.5), C 2.6 (0.5)	EX 36 (10), C 37 (11)	Balance, motor agility exercises combined with cognitive or motor tasks.	On	No intervention.	10	3 X 60’	GAITRITE	SPEED, STEP LENGTH, CADENCE
**Duncan and Earhart 2012**	USA	26	EX 2.6 ± 0.1, C 2.5 ± 0.1	EX 44.5 ± 2.3, C 48.0 ± 1.8	Community-based tango program including walking while performing cognitive task (naming).	Off	No intervention.	48	2 X 60’	GAITRITE	SPEED
**Geroin 2018**	Italy, Netherlands, Belgium	56	EX 2.3 (0.5) , C 2.3 (0.5)	EX 32.25 (11.73)	Concurrent practice of gait exercises and cognitive tasks.	On	Separate practice of gait exercises and cognitive tasks.	6	2 X 40’	GAITRITE	STRIDE LENGTH, CADENCE, NUMBER OF FALLS
**King 2020**	USA	25	EX 2.4 (0.7), C 2.6 (0.9)	EX 44.9 (11.8), C 50.0 (14.3)	Walking while performing different cognitive task	On	Education on: the care team, sleep, mood, nutrition, medication, stress management.	6	3 X 80’	WEARABLE SENSORS: 8 IMU STERNUM, LUMBAR SPINE, WRISTS, SHINS, FEET	DT COST ON SPEED
**Mirelman 2016**	Israel, Netherlands, Belgium, Italy	66	EX 2.44 ± 0.46	EX 32.33 ± 14.65	Walking on treadmill in a virtual environment engaging cognitive domains.	On	Walking on treadmill.	6	3 X 45’	ZENO WALKWAY	SPEED, STEP WIDTH

**Pelosin 2022**	Italy, Israel, Netherlands, Belgium, UK	24	EX 2.5 (0.4), C 2.4 (0.5)	EX 30.7 (9.2), C 32.3 (14.6)	Walking on treadmill in a virtual environment engaging cognitive domains.	On	Walking on treadmill in a virtual environment engaging cognitive domains.	12	3 X 45’	WEARABLE SENSORS: 3 ACCELEROMETERS UNDER HEELS AND ON THE BACK (L5) + RECORDING PLATFORM	SPEED, STRIDE LENGTH
**Penko 2019**	USA	10	EX 2.4 (0.5), C 2.2 (0.4)	EX 34.5 (10.3), C 38.2 (12.9)	Walking training with concurrent cognitive training.	On	Walking training and consecutive cognitive training.	8	3 X 45’	\	FALL FREQUENCY, AVERAGE STEPS PER DAY
**Rosenfeldt 2019**	USA	10	EX 2.4 (0.5), C 2.2 (0.4)	EX 35 (10), C 38 (13)	Walking training with concurrent cognitive training.	On	Walking training and consecutive cognitive training.	8	3 X 45’	PHOTO-GRAMMETRY: VICON	SPEED, STEP LENGTH, CADENCE
**Sarasso 2021**	Italy	12	EX 2.4 (0.4), C 2.4 (0.4)	EX 26.27 (9.88), C 28.83 (8.47)	Gait/balance + cognitive training: AOT-MI combined with practicing the observed-imagined exercises.	On	Gait/balance + cognitive training watching landscapes.	6	3 X 60’	PHOTO-GRAMMETRY: BTS	DT TURNING SPEED, DT COST ON TURNING SPEED
**Strouwen 2017**	Netherlands, Belgium	56	EX 2.3 (0.5), C 2.3 (0.5)	EX 32.25 (11.73)	Concurrent practice of gait exercises and cognitive tasks.	On	Separate practice of gait exercises and cognitive tasks.	6	2 X 40’	GAITRITE	SPEED, NUMBER OF FALLS
**Valenzuela 2020**	Spain	23	EX 2.6 (0.6), C 2.5 (0.7)	\	Consecutive and simultaneous cognitive task while performing walking exercises.	On	Single task walking exercises.	10	2 X 60’	PHOTO-GRAMMETRY: KINESCAN/IBV	SPEED, STRIDE LENGTH, CADENCE, STEP WIDTH
**Wallén 2018**	Sweden	47	EX 2.5 (0.5), C 2.6 (0.5)	EX 36 (10), C 37 (11)	Balance, motor agility exercises combined with cognitive or motor tasks.	On	No intervention.	10	3 X 60’	GAITRITE	SPEED, STEP LENGTH

**Wong-Mak 2015**	China	41	EX 2.5 (0.3), C 2.4 (0.3)	EX 26.9 (10.4), C 31.3 (11.1)	Perturbation-based walking training with cognitive daily tasks (pulling or pushing doors, exiting or entering escalator or elevator, fast walking).	On	Upper limb training.	8	1 X 120’	STOPWATCH	SPEED, BALANCE CONFIDENCE
**Yang 2019**	Taiwan	6	EX 2.0 (1.6–2.6), C 1.5 (0.9–2.4)	\	Cognitive and motor tasks during different walking conditions.	On	General gait training: walking forward, backward, S-shape route, obstacles.	12	3 X 30’	GAITRITE	SPEED, STRIDE LENGTH, CADENCE, BALANCE CONFIDENCE

Abbreviations

EX: Experimental group; C: Control group; DT: Dual-task

Table S2. GRADE, secondary outcomes.

Certainty assessment							№ of patients		Effect		Certainty	Comments
№ of studies	Study design	Risk of bias	Inconsistency	Indirectness	Imprecision	Other considerations	Dual-task gait training	Single-task gait training	Relative (95% CI)	Absolute (95% CI)		
**Dual-task cost on gait speed**												
3	randomized trials	serious (a)	not serious	Very serious (b)	not serious	none	44	6	-	**0.99 standard deviations higher** (0.10 to 1.89 higher)	⊕◯◯◯ Very Low	Very large effect favoring dual-task relative to single-task gait training in reducing dual-task cost on gait speed in people with PD
**Balance confidence**												
4	randomized trials	not serious	not serious	Very serious (c)	Serious (d)	none	84	6	-	**0.63 standard deviations higher** (-0.27 to 1.53 higher)	⊕◯◯◯ Very Low	Large, non significant effect favoring dual-task relative to single-task gait training in improving balance confidence in people with PD
**Quality of life**												
7	randomized trials	not serious	serious (e)	Very serious (c)	not serious	none	247	81	-	**0.55 standard deviations higher** (0.02 to 1.08 higher)	⊕◯◯◯ Very Low	Large effect favoring dual-task relative to single-task gait training in improving quality of life in people with PD

CI: confidence interval.

Explanations

a. Overall risk of bias for the outcome judged to have some concerns
b. Different outcome measures were used across studies assessing this outcome. Furthermore, one comparison group was used for the other studies
c. One comparison group was used for the other studies
d. Imprecision, wide non-significant confidence interval
e. I squared=86%
